# Somatic Cell Number, Physicochemical, and Microbiological Parameters of Raw Milk of Goats During the End of Lactation as Compared by Breeds and Number of Lactations

**DOI:** 10.3389/fvets.2021.694114

**Published:** 2021-09-03

**Authors:** Rreze M. Gecaj, Flutura C. Ajazi, Hysen Bytyqi, Blerta Mehmedi, Hazir Çadraku, Muharrem Ismaili

**Affiliations:** ^1^Department of Animal Husbandry, Faculty of Agriculture and Veterinary, University of Prishtina, Prishtina, Kosovo; ^2^Department of Food Science and Biotechnology, University for Business and Technology-Higher Education Institution, Prishtina, Kosovo; ^3^Institute of Microbiology, Vifor Pharma, Glattbrugg, Switzerland

**Keywords:** goat's milk, native Red, somatic cells, Alpine, mesophilic bacteria

## Abstract

This study was aimed for the evaluation of somatic cell count (SCC), physicochemical, and microbiological parameters during the end of lactation in the raw milk of Alpine and native Red goat breed. In the experiment, 102 milk samples from Alpine and native Red goats were included. Two different groups within the same breed were analyzed: a group consisting of animals in their first lactation and the second group consisting of animals from the fifth lactation. The milk samples were individually and daily collected during late lactation for three consecutive weeks, and milk fat, protein, lactose, SCC, and total bacteria with enterobacteria were assessed. Fresh milk of goats from late lactation period had a number of somatic cells (SC) within the expected value with log10 of 5.8–6.18 cells/ml for the compared groups. In both breeds, the total mesophilic bacteria were fewer in numbers, however, in the native Red goat, a larger population of such bacteria was enumerated. The number of coliforms and enterobacteria was below 100 cfu/ml. In the current study, we were able to show a significant difference among the studied breeds depending on lactation and season for fat (*p* = 0.002), but not for lactose and protein content. A positive correlation for total protein (TP), lactose, and fat as well as for lactose and SCC was found in the native Red goat breed. In the Alpine goat breed, a strong positive correlation (0.821^**^) was found for lactose and enterobacteria count (EC). In conclusion, these findings evaluate different goat milk parameters during late lactation period and provide an indirect measure to monitor goat mammary gland health for both breeds.

## Introduction

Milk is the only food for mammals in their first period of life. This nutritive fluid is secreted from the mammary gland of different animals such as cows, sheep, goats, buffalo, as well as humans. The unprocessed milk components are related to its composition and are slightly different between animal species and are influenced by the animal health condition, animal age, breed, and lactation period (1). In developing countries, goat milk is an essential source of food for home consumption and in addition, it contributes to the family economy, while in the developed countries, goat milk products such as cheese and yogurt are mainly consumed (2).

The production of goat milk is considered to be much higher than the official statistics, because of the large amount of unreported home consumption, especially in the developing countries (2). According to FAOSTAT (3), goat milk production contributed to the global milk production in 2010 with 2.4%. In the Mediterranean area and the Middle East, as well as Kosovo, goat breeding has a century-long history. These small ruminants have been used for milk production and milk products, as well as for meat, skin processing, and cosmetics (2).

Goat production in Kosovo consists of 27,197 heads (4), including all categories and breeds. However, ~two-thirds of this number is represented by the strains of the Balkan breed (spotted, white, yellowish, and red strain) bred as a dual-purpose breed (meat and milk) (5). Native Red strain is an autochthon goat, a very spread animal all over the Kosovo regions. It is characterized by strong body conformation that suits well at minimum levels of management and semi-extensive production. This goat strain is well-adapted to natural grazing for a period of 9 months (March–November). Parturition mostly occurs during the winter period. As in other strains of this breed, native Red goat milk production is ~80–130 kg/milk over the 180–200 lactation days (5).

The Alpine breed that constitutes about one-third of the total goat number in Kosovo was imported after the Kosovo war at the beginning of 2000s and is characterized by a higher milk production (unpublished data). The Alpine goats used in the present study were born and raised under Kosovo environment production.

Although, there is no scientific evidence of native Red and Alpine goat mammary gland infections or any other diseases, according to farmers, there is a minimum of such incidents which make them quite resistant and adaptive to these environment conditions (4).

Goat milk differs with some physicochemical properties, such as fatty acids, lactose, size of the fat globule, enzymes, minerals, and vitamins from cows and sheep milk. The milk composition depends on many factors, such as breed, season, stage of lactation, nutrition, and individual traits (6–9).

While the composition of cow milk is stable, sheep and goat milk composition changes by seasons, which reflect the physiological status of goats (10, 11). It is shown that the protein, fat, and mineral content increases, while lactose decreases by the end of lactation (2). Moreover, goat milk contains a greater amount of Ca, P, Mg, and Cu than cow milk under identical environmental conditions (12).

Other authors also reported that the week of lactation affected significantly all major and trace minerals in milk and the greatest contents for almost all the minerals were observed at the end of lactation (13). Despite the nutritive values of goat milk, the consumers refuse to take goat's milk-based products, because of their characteristics flavor. Current evidence suggests that the presence of probiotic bacteria (lactic acid bacteria) in goat milk is very important for improving the aroma of goat milk-based products and for the enhancement of their nutritional value (14, 15).

Goat milk is richer on short and medium chain FAs with 6–10 carbon atoms compared to cow milk, and their milk flavor is especially influenced by the C7 short-chain fatty acids, which is characteristic for the goat (13, 16). The size of fat globules in goat milk is smaller than in cow milk, which may make it more easily digestible (17). Goat milk is a poor source of folate in contrast with cow milk (18), and contains proteins with different genetic polymorphism than cow milk, resulting in low allergenicity (19). The amino acid composition in proteins of goat and cow milk was found to be also different and the level of six essential amino acids was higher in goats. Among them, cysteine showed the highest difference and was found to be beneficial for the treatment of malnutrition syndrome in mice studies (20, 21).

The presence of the oligosaccharides sialic acid in goat milk is many times higher than in cow milk (17). The higher contents of oligosaccharides may promote bifidobacterial growth in the gut of new-born and play a major role in brain development (19).

Somatic cell count (SCC) in raw milk is another important measure successfully used in dairy cattle to monitor the bacterial infections (Mastitis). Mastitis has an impact on the economics of milk production and can decrease its quality and technological properties (22). Subclinical mastitis is critical, because it can lead to the reduction of milk production and milk quality that is particularly important when unpasteurized milk is used for cheese production (23). Subclinical mastitis in goat is common and is mainly caused by Staphylococci (CNS) and *Staphylococcus aureus* (*S. aureus*) bacteria (24). The number of somatic cells (SC) is the most commonly used indicator for milk gland health in dairy cow, sheep, and goat, but unfortunately, the number of SC is difficult to interpret in goats (25, 26). Compared to sheep and cows, SC count in goat milk is also relatively high in healthy milk glands and increases throughout lactation (27).

Milk and cheese yield in goats were shown to be reduced and negatively correlate with the bacterial standard plate count (SPC) (27). Thus, the evaluation of SPC in raw milk combined with other quality parameters has been shown as a good indicator of mammary gland infection (mastitis) in different dairy breeds of goats. With this measure, it is also possible to draw conclusions in regard to mammary gland susceptibility for bacterial infection as the number of lactations increases and the animals become more prone to mastitis by the end of lactation season (28, 29).

Therefore, this study aims to evaluate the SCC, physicochemical, and microbiological parameters during the end of lactation season in the raw milk of a goat. In order to examine the relationship between these parameters, milk samples were taken from two goat breeds Alpine and native Red goats in their first and fifth lactation.

## Materials and Methods

### Sampling and Animals

**Figure 1:**
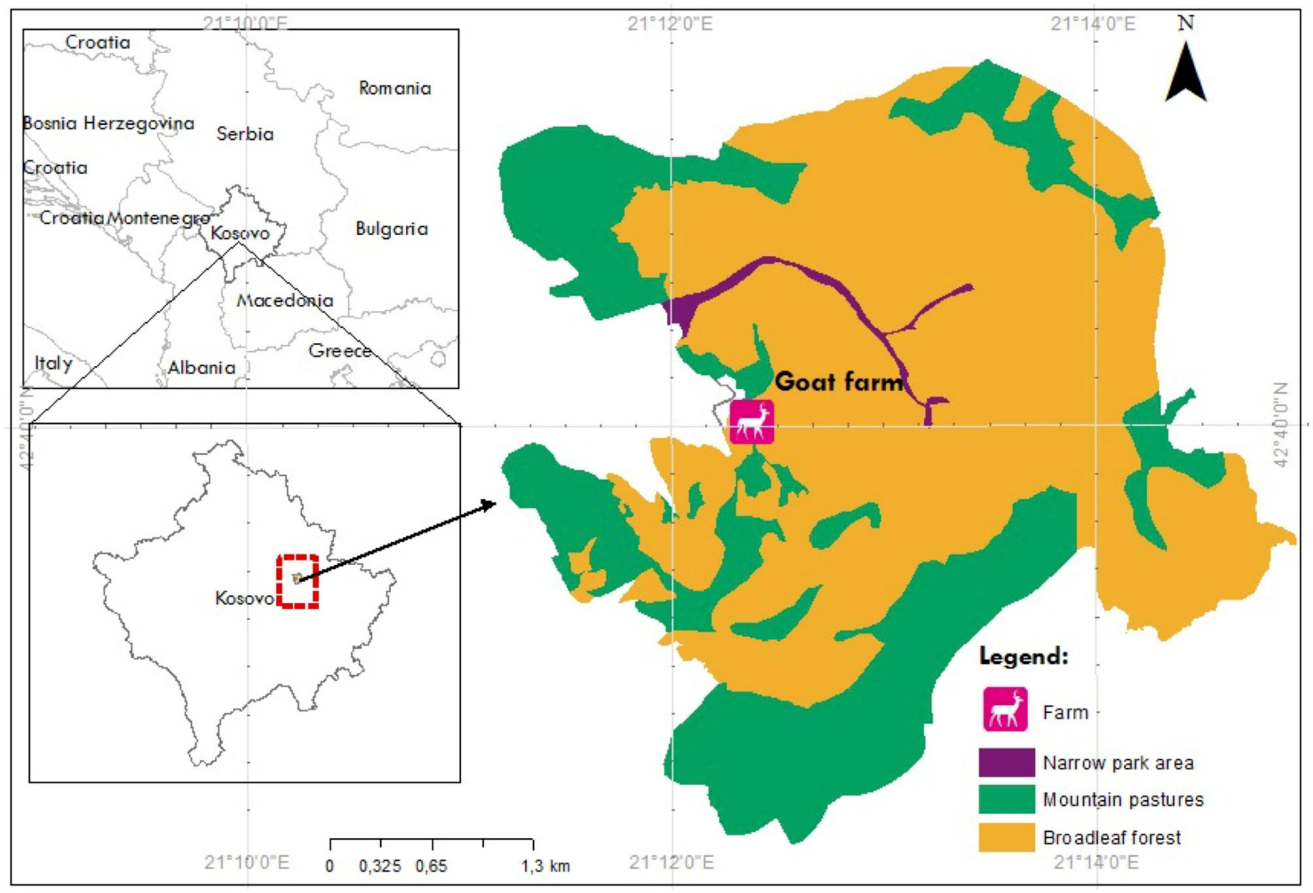
The geographical location of goat farm included in the study.

A total number of 102 samples from Alpine and native Red goad breeds, collected in the region of Pristina, Kosovo (Figure 1) were included in the experiment. The Alpine and native Red dairy goats were housed in a family farm consisting of 250 goats of Alpine and native Red goat. The approximal weight of animals was 38–40 kg for the young group and 45–50 kg for the old group of animals. Within the same breed, two different groups were analyzed: a group of old goats in their fifth lactation (old, *n* = 10) and a young group in their first lactation (young, *n* = 10). The lactation lasts ~180–200 days (5). Sampling was carried out for three consecutive weeks during late lactation, from October to November 2018.

Milk samples were collected in the morning, from each animal, 50 ml of milk were aseptically collected into three individual containers, one for each of the planned analysis: SCC, physicochemical parameters, and microbiological. All samples were transported using cooling boxes. Microbiological analysis was carried out within 3 h after milk sampling.

### Goat Management Practice

Normally, goats are kept outdoors most of the time during the whole year. During the grazing period, feeding is dependent on the feed taken outside and a small portion of homemade concentrate feed (mostly corn grain). Stable is of average hygienic condition where manure is cleaned twice a year in most of the goat farms. Milking occurs 100% by hand (manually) twice a day, before grazing (morning) and after grazing (evening), specifically. Data recording options are not available in the goat farms in Kosovo. The climate in Kosovo is typically semi-continental characterized by hot summer and cold winter time. During the experiment, the average air temperature was 6.64°C (max. 25.83°C, min. −8.46°C).

### Somatic Cell Count

For all raw goat milk samples, the SC number was determined using a Somatic cell counter (MT05, Slovakia). All samples were fresh during the procedure and were softly shaken. Sample temperature during the measurement was 36°C.

The method determines the SCC based on the change of milk sample viscosity. For the determination of SCC, 10 ml of milk sample are mixed (with a syringe that is a part of the accessories of MT05) with 5 ml of 20% S4 reagent. The mixture causes a change in the viscosity of the milk proportional to the quantity of SC. The MT05 instrument is similar to a Höppler viscometer and the measurements were performed automatically by the device electronics within a measuring range 10 × 10^3^ to 2 × 10^6^ ml^−1^ (30).

### Bacterial Count

The raw milk was serially diluted (1:10) in Peptone water solution and all samples were plated for bacterial enumeration according to the pour plate method used by Ajazi et al. (31). Briefly, 1 ml aliquots of the diluted samples were inoculated directly into the molten media. Certain culture media were weighed on an analytical scale, distilled water was added according to the factory prescription and mixed in the magnetic stirrer hot plate. After that culture media were sterilized in an autoclave for 15 min in 121°C, in pressure 1.5 atm.

The total number of colonies (TNC) were cultured in milk plate count agar (MPCA; Liofilchem, Italy) and counted after 72 h of incubation at 30°C. Coliforms and enterobacteria were grown on MacConkey agar (MCA; Biolife, Italy) and were counted after 48 h of incubation at 37°C. We proceeded with the enumeration of colonies in all plates consisting of between 30 and 300 colonies. Plate counts were log transformed before statistical analysis.

### Physicochemical Parameters

Protein, fat, and lactose content were determined from raw milk samples using an IR (infra-red) beam-based device LactoScan (Milkotronic Ltd., Bulgaria).

After the collection, all milk samples were frozen with sodium azido (Merck, Germany) as conservatives and transported in refrigerated conditions (4°C) for analysis.

Before measurement, all milk samples were warmed up to 10–15°C and stirred according to manufacture instructions (LactoScan, Milkotronic Ltd., Bulgaria).

To exclude any interference from previous samples, the device was cleaned with warmed (60°C) NaOH 1.5%, 2% HCl, and several washing-steps with distilled water.

### Statistical Analysis

All experimental data are presented as the mean of 3 weeks ± SEM. However, for a detailed comparison of the results, weeks were also compared individually. Statistical significance was determined using two-way ANOVA, and differences between means were compared by Holm-Sidak's test for multiple comparisons. Pearson's correlation coefficient is calculated using SigmaStat V3. The results were considered significant if *p* < 0.05.

## Results

### Total Protein, Fat, Lactose, and Somatic Cell Count

**Figure 2:**
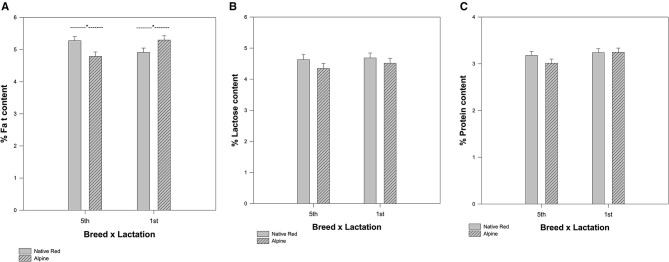
The difference in mean values among the breeds depending on lactation season for **(A)** fat, **(B)** lactose, and **(C)** protein content. The interaction between breed and lactation is significant for fat (*p = 0.002), but not for lactose (*p* = 0.716) and protein (*p* = 0.328).

The mean values for protein, fat, and lactose content in the milk of native Red and Alpine goat breed were different and the measured constituents were increased over the 3 weeks during late lactation (Supplementary Table 1). The milk composition varied significantly between Alpine and native Red goat breed in their fifth lactation season, while the composition of milk was very similar between Alpine and native Red goats in their first lactation season. In the milk for the native Red goat (fifth lactation season), we measured significantly more fat (*p* = 0.002) compared with the Alpine goats (Figure 2A). However, the interaction between breed and lactation was not significant for lactose (*p* = 0.716) and protein (*p* = 0.328) (Figures 2B,C). Protein content ranged from 2.8 ± 0.06 to 3.5 ± 0.2% and lactose content ranged from 4.08 ± 0.1 to 5.4 ± 0.3%. A higher level of milk lactose was measured in the last week of lactation season for native Red goat breed in their fifth lactation. Somatic cell count did not change significant changes during weeks, at the late lactation season (Supplementary Table 1). Protein content of raw milk for both breeds in their first location season, tend to be stable during the weeks and was in the range of 2.9 ± 0.05 to 3.7 ± 0.2%. A similar trend was found for lactose 4.02 ± 0.2 to 5.3 ± 0.1% (Supplementary Table 1).

**Figure 3:**
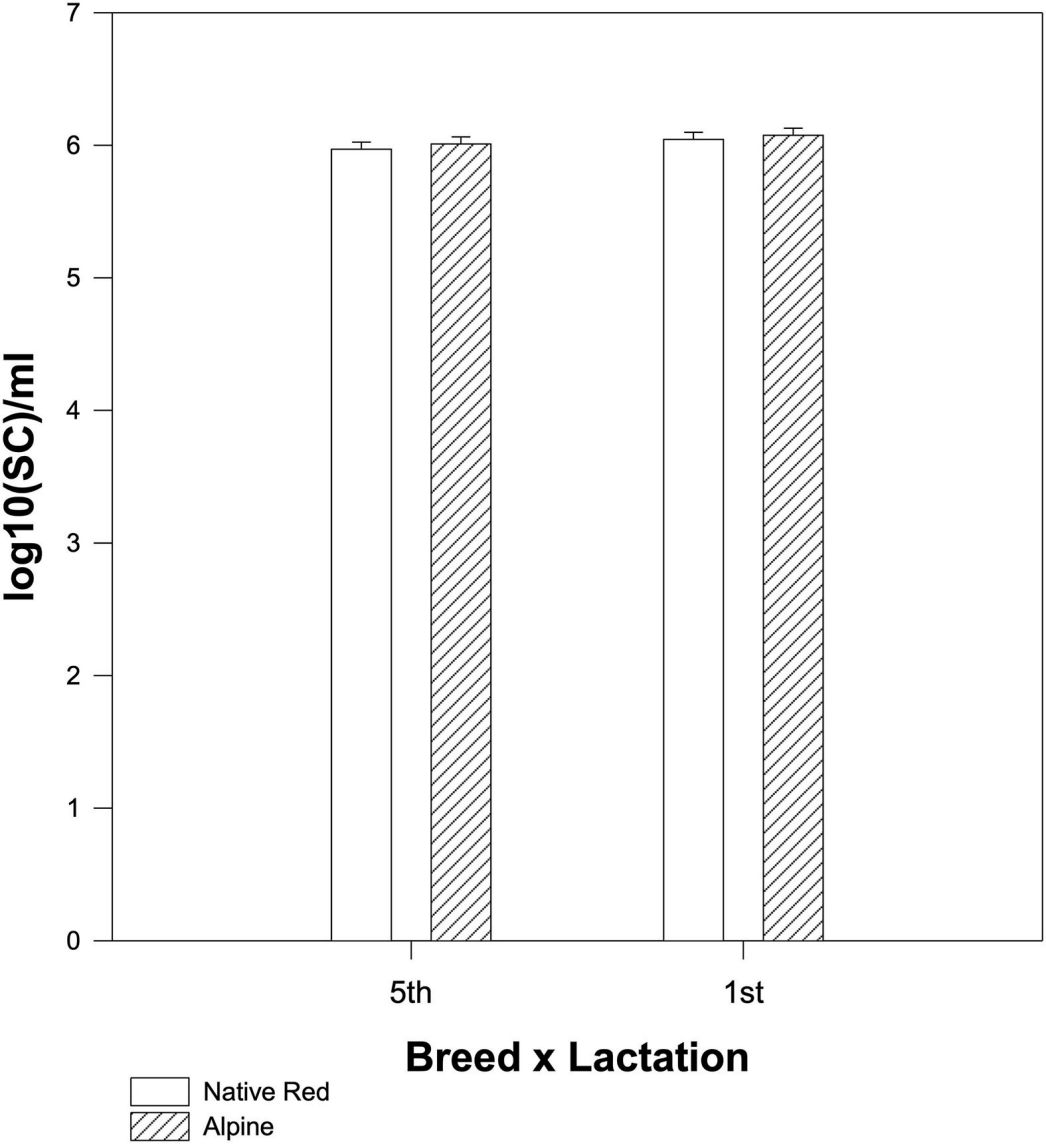
The difference in mean values among the breeds depending on the lactation season for the somatic cell counts (SCCs). The interaction between breed and lactation was not significant for SC (*p* = 0.823).

The native Red and Alpine goat breed during the first lactation season had a slightly higher number of SC/ml in raw milk but not significant (*p* = 0.306) compared to native Red and Alpine goats from the fifth lactation season (Figure 3).

### Microbiological Results

The results from microbiological analysis for the presence of total mesophilic bacteria and enterobacteria and coliforms in goat milk during the late lactation season are presented in Table 1. The TNC in the first week that were measured in all groups of dairy goats ranged between 2.9 × 10^2^ and 4.8 × 10^2^ cfu/ml and there was a significant lower number of colonies in the native Red dairy goats from the first^t^ lactation season (Table 1, *p* = 0.03). In the second week, TNC varied between 2.5 × 10^2^ and 3.4 × 10^2^ cfu/ml, but was not significant between breeds. Enterobacter and coliform count in all milk samples were found to be lower than 100 cfu/ml.

**Table 1:** The total number of colonies, Enterobacter and coliform (cfu/ml) of goat milk samples at the second week and third week of lactation.

	**Alpine, 5^th^** **lactation**		**Native Red, 5^th^** **lactation**		**Alpine, 1^st^** **lactation**		**Native Red, 1^st^** **lactation**	
**Week**	**I**	**II**	**I**	**II**	**I**	**II**	**I**	**II**
TNC^†^	4.8 × 10^2^ ± 0.5	3.1 × 10^2^ ± 1.2	4.5 × 10^2^ ± 0.7	3.0 × 10^2^ ± 0.2	3.7 × 10^2^ ± 0.8	2.5 × 10^2^ ± 0.4	2.9 × 10^2^ ± 0.8^*^	3.4 × 10^2^ ± 0.8
EC^‡^	<100	<100	<100	<100	<100	<100	<100	<100

† *Total number of colonies*.

‡ *Enterobacter and coliform*.

* *p < 0.05*.

### Correlation of the Measured Parameters

The correlation coefficients among different parameters for the analyzed breeds and lactation seasons are summarized in Table 2. A significant positive correlation for total protein (TP) and fat (0.633^*^), as well as TP and lactose (0.682^**^) were found in the native Red goat breed in their first and fifth lactation season, respectively (Tables 2A,B). In addition, the native Red goats from their fifth lactation season had a significant positive correlation for lactose and SCC (0.531^*^). In the Alpine goat breed from first lactation, a strong positive correlation (0.821^**^) was found for lactose and enterobacteria count (EC). Total protein were also strongly positive correlated to lactose and fat content in Alpine goat breed from both the first and fifth lactation season (Tables 2C,D). We found a positive correlation of SC with TP, fat, and lactose except for the native Red goat breed in the first lactation in which SCC had a negative correlation with fat (−0.056).

**Table 2:** Correlation coefficients among different milk variables in Alpine and native Red goat breed.

**Parameters**	**TP**	**Fat**	**Lac**	**SCC**	**TNC**	**EC**
**(A) Correlation Coefficients Among Milk Variables for the First Lactation of native Red Goat Breed**						
TP	1					
Fat	−0.031	1				
Lac	0.633^*^	0.12	1			
SCC	0.087	0.063	0.355	1		
TNC	0.064	−0.056	−0.482	0.119	1	
EC	0.482	0.328	0.45	−0.419	−0.528	1
**(B) Correlation Coefficients Among Milk Variables for the Fifth Lactation of the native Red Goat Breed**						
TP	1					
Fat	0.682^**^	1				
Lac	0.958	0.6^*^	1			
SCC	0.387	−0.056	0.531^*^	1		
TNC	−0.324	−0.468	−0.377	−0.137	1	
EC	0.218	0.401	0.329	0.393	−0.447	1
**(C) Correlation Coefficients Among Milk Variables for the First Lactation of the Alpine Goat Breed**						
TP	1					
Fat	0.631^**^	1				
Lac	0.857^**^	0.664^**^	1			
SCC	0.307	0.346	0.392	1		
TNC	0.581	−0.067	0.606	−0.376	1	
EC	0.632	−0.345	0.821^**^	0.36	0.382	1
**(D) Correlation Coefficients Among Milk Variables for the Fifth Lactation of the Alpine Goat Breed**						
TP	1					
Fat	0.513^*^	1				
Lac	0.895^**^	0.325	1			
SCC	0.209	0.045	0.192	1		
TNC	0.567	0.158	0.249	0.373	1	
EC	0.034	−0.304	0.438	−0.0001	0.336	1

* *p < 0.05 and*

** *p < 0.01*.

In the native Red goat breed from the first lactation, TP had a negative correlation to fat (−0.031) but positive correlation to other study parameters, fat had a negative correlation to TNC (−0.065), but positive correlation to other parameters and lactose had a negative correlation to TNC (−0.482), but positive correlation to SCC and EC (Table 2A).

In the native Red goat breed from the fifth lactation, fat had a negative correlation to SC (−0.056), and TNC (−0.468) but a positive correlation to lactose and EC, lactose had a negative correlation to TNC (−0.137) but positive correlation to SCC and EC (Table 2B).

In the Alpine goat breed from the first lactation, fat had a negative correlation to TNC (−0.067) and EC (−0.345), but a positive correlation to other parameters (Table 2C).

In the Alpine goat breed from the fifth lactation, fat had a negative correlation to EC (−0.304), but a positive correlation to lactose, SCC, and TNC (Table 2D).

## Discussion

To the best of our knowledge, this is the first study analyzing the physicochemical and microbiological properties of raw milk of goats in the region of Kosovo. The measured physicochemical parameters in milk (fat, protein, and lactose) in general show a normal trend for goat milk. However, we found a tendency for higher milk fat content, that could be explained by the feeding practice in Kosovo mostly consisting of high cellulose, the lactation season (late lactation), and other managerial characteristics (32, 33). According to Kovácová et al. (34), fat content tends to be significantly higher in early and late lactation than in middle lactation in goat milk (34). In addition, TP was increased in late lactation for both breeds, which is similar to Kuchtik et al. (35) and can be related to milk volume reduction (36). Similar trend is shown in other studies in which protein content was in the range of 3.26 ± 0.65 to 4.01 ± 0.67% from early lactation to late lactation (34). The content of lactose found in the current study is in accordance with Rolinec et al. (37) that measured 4.73% lactose in goat milk.

The counting of SC in raw milk of Alpine and native Red goats, as well as the detection of total mesophilic bacteria and Enterobacteriaceae were also analyzed. The number of SC in our study were within the allowed legislative requirements for raw goat milk [mean log10 (SC) varying in the range of 5.41–6.18 cells/ml]. According to Moroni et al. (38), who conducted goat milk quality monitoring program in Northern Italy, similar results for SC number was observed in positive bacteriological samples (5.6 cells/ml), while for negative bacteriological samples, these authors reported a significantly lower number of SC (3.9 cells/ml). However, in a study performed by Podhorecka et al. (39), a higher number of SC were counted in goats free of mastitis-causing bacteria (MCB), highlighting again the need for determination of standards for the number of SC, which are difficult to interpret in goats (25, 27).

The results of our research show a trend of increasing SC number during the late lactation season in all groups. However, due to the high variation in the number of SC among the individual animals, this trend is not significant. For this reason, further research is needed consisting of a higher number of animals that would be monitored for a longer period, for example, throughout lactation. The SC in raw milk of Alpine and native Red goats have been in positive correlation with proteins, which are in accordance with the study of Kovácová et al. (34), who found the highest content of TP in the same month when the number of SC was higher SC in raw goat milk.

As a product that spoils easily if not stored in the right conditions (1–7°C), microbiological parameters of milk are mandatory to be performed (40). The present study results show that the total number of mesophilic bacteria and Enterobacteriaceae in coliforms (EC) was within the allowable values for raw milk (<100 cfu/ml) and in accordance with the national raw milk quality standards for bacterial content (AG, MA-NR.20/2006).

However, the number of mesophilic bacteria in raw goat milk in the current study was lower compared to the findings of other authors (41). According to Dalzini et al. (42), mesophilic bacteria and Enterobacteriaceae in raw goat milk used for the production of *Formaggelle di capra* cheese were higher than the current study results. Although bacteriological values in fresh milk serve to assess the association between possible mammary gland infections and SCCs, no correlation has been observed between these two parameters in the study. Other authors also found no correlation between SCC and mastitis in goat and sheep milk, contrary to cow milk (34, 43). According to another study from a mixed linear model, SC concentrations are expected to increase with age and in the presence of bacteria, among which *S. aureus* infections were associated with higher SC numbers (38). Since in the present study, we counted the total mesophilic bacteria in general and not in particular *S. aureus*, no conclusion can be drawn if the presence of *S. aureus* is associated with a high SC count.

A study performed by Ying et al. (28) found a significant positive correlation between TNC and SCC for Alpine dairy goats in early lactation but not in late lactation, which is in accordance with the present study. During the monitoring period in the current study, TNC was <2.73 cfu/ml which correlates well with findings from other authors (41). The number of EC in milk samples for all groups of goats in our study ranged between 1.0 and 1.95 cfu/ml that is similar to 1.87 cfu/ml found by Lai et al. (44). In addition, TP, fat, and lactose showed a positive correlation with EC, whereas SCC and TNC had a negative correlation with EC.

The TNC showed a negative correlation with TP, fat, lactose, and SCC in native Red goats fifth lactation, while EC positively correlated with these parameters except for TNC (−0.447). On the other hand, for Alpine goats in their fifth lactation, a positive correlation with TNC was measured for all analyzed parameters. Enterobacteria count showed a positive correlation with TP, lactose, and TNC but a negative correlation with fat (−0.304) and SCC (−0.0001), which is different compared to native Red dairy goats from the same lactation season.

For Alpine goats in their first lactation season TNC showed a positive correlation with TP and lactose, but a negative correlation with fat (−0.067) and SCC (−0.376). Among all these parameters, EC was highly correlated with lactose (0.821^**^), which can be expected considering that EC are a lactose-utilizing genus (45). The TNC in the first lactation season for native Red goats show a positive correlation with TP (0.064) and SCC (0.119), but a negative correlation with fat (−0.056) and lactose (−0.482). A similar result has been reported by Ying et al. (28) in goat milk of Saanen dairy.

The EC from goat milk observed in the study was substantially lower than reported by other authors (42, 46). All analyzed microbiological and physicochemical parameters of goat milk are shown to be crucial for qualitative and safe cheese production under small scale farms (12, 39, 42, 47).

## Conclusions

The finding of this study provides information for locally raw goat milk related to SCC, microbiological, and physicochemical parameters during late lactation season of native Red and Alpine goats. In the current study, we were able to show a significant difference among the studied breeds depending on the lactation and season for fat, but not for lactose and protein content.

However, this study shows no difference in the number of SCC between the first and fifth lactation season in native Red and Alpine goats. The TNC and EC in goat milk were within the allowed legislative required limits and this can be attributed to the management practices. Therefore, based on these data, we can suggest that the milk was microbiologically safe for the production of milk products, especially for artisan and homestead cheese.

## Data Availability Statement

The original contributions presented in the study are included in the article/Supplementary Material, further inquiries can be directed to the corresponding author.

## Ethics Statement

Ethical review and approval was not required for the animal study because the study doesn't implicate any animal experiments. We used goat milk samples that are collected by hand during the routine daily animal milking procedure.

## Author Contributions

RG and FA: conceptualization, methodology, formal analysis, and writing—original draft preparation. RG, FA, HB, BM, and MI: writing. RG, FA, and HÇ: visualization. All authors contributed to the article and approved the submitted version.

## Conflict of Interest

The authors declare that the research was conducted in the absence of any commercial or financial relationships that could be construed as a potential conflict of interest.

## Publisher's Note

All claims expressed in this article are solely those of the authors and do not necessarily represent those of their affiliated organizations, or those of the publisher, the editors and the reviewers. Any product that may be evaluated in this article, or claim that may be made by its manufacturer, is not guaranteed or endorsed by the publisher.
