# Supplementary material for: Somatic Cell Number, Physicochemical, and Microbiological Parameters of Raw Milk of Goats During the End of Lactation as Compared by Breeds and Number of Lactations
Source: Front Vet Sci. 2021 Sep 3;8:694114. doi: 10.3389/fvets.2021.694114 (PMC8446551; doi:10.3389/fvets.2021.694114)
Supplement: Supplementary file 1 [file Table_1.DOCX]

Supplementary Material

**Supplementary Table S1.** Comparison of milk fat, milk protein, lactose and SCC between Alpine and native Red goats during the last three weeks of lactation season.

|  | Alpine, lactation 5^th^ | | | Native Red, lactation 5^th^ | | |
| --- | --- | --- | --- | --- | --- | --- |
| Week | I | II | III | I | II | III |
| Milk fat (%) | 5.1$\pm0.0$ | 4.2$\pm0.1$ | 5.04$\pm0.3$ | 4.8$\pm0.2$ | 5.11$\pm0.0$****** | 5.9$\pm0.0$***** |
| Milk protein (%) | 2.9$\pm$0.1 | 2.8$\pm$0.06 | 3.2$\pm$0.09 | 2.9$\pm$0.1 | 3.02$\pm0.0$***** | 3.5$\pm0.2$ |
| Lactose (%) | 4.09$\pm$0.3 | 4.08$\pm$0.1 | 4.8$\pm$0.2 | 4.2$\pm$0.3 | 4.3$\pm$0.2***** | 5.4$\pm$0.3***** |
| log (SCC) | 6.08$\pm$0.05 | 6.1$\pm$ 0.03 | 5.9$\pm0.19$ | 5.9$\pm$0.1 | 5.9$\pm0.1$ | 6.1$\pm0.04$ |

|  | Alpine, lactation 1^st^ | | | | Native Red, lactation 1^st^ | | | |  |
| --- | --- | --- | --- | --- | --- | --- | --- | --- | --- |
| Week | | I | II | III | | I | II | III | |
| Milk fat (%) | | 5.1$\pm$0.1 | 5.0$\pm$0.0 | 5.7$\pm$0.2 | | 4.9$\pm$0.1 | 4.6$\pm$0.2 | 5.2$\pm$0.3 | |
| Milk protein (%) | | 2.9$\pm$0.05 | 3.1$\pm$0.2 | 3.7$\pm$0.2 | | 3.2$\pm$0.1***** | 3.2$\pm$0.07 | 3.4$\pm$0.1 | |
| Lactose (%) | | 4.02$\pm$0.2 | 4.2$\pm$0.3 | 5.3$\pm$0.1 | | 4.5$\pm$0.1 | 4.6$\pm$0.1 | 4.9$\pm$0.1 | |
| log (SCC) | | 6.1$\pm$0.04 | 6.1$\pm$0.03 | 6.1$\pm$0.3 | | 6.1$\pm$0.05 | 6.1$\pm$0.3 | 6.1$\pm$0.1 | |

**p<0.01; *p<0.05
